# Aortic Elastic Properties and Albumin-Based Inflammatory Indices in Dyspneic Third-Trimester Pregnant Women: A Prospective Observational Study

**DOI:** 10.3390/biomedicines14020483

**Published:** 2026-02-22

**Authors:** Birsen Ertekin, Hatice Eyiol, Azmi Eyiol, Fatih İkiz, Rukiye Ozcelik Tepe

**Affiliations:** 1 Department of Emergency, Beyhekim Training and Research Hospital, 42130 Konya, Türkiye; sultanmehmet01@hotmail.com; 2 Department of Anesthesia and Reanimation, Beyhekim Training and Research Hospital, 42130 Konya, Türkiye; haticerkan42@hotmail.com; 3 Department of Cardiology, Beyhekim Training and Research Hospital, 42130 Konya, Türkiye; azmieyiol@yahoo.com; 4 Department of Obstetric Gynecology, Beyhekim Training and Research Hospital, 42130 Konya, Türkiye; drrozcelik@gmail.com

**Keywords:** dyspnea in pregnancy, aortic elastic properties, aortic stiffness, albumin-based inflammatory indices, C-reactive protein-to-albumin ratio, echocardiography

## Abstract

**Background:** Dyspnea is a frequent complaint during pregnancy and is often considered a benign physiological finding; however, it may also reflect underlying subclinical cardiovascular alterations. Pregnancy-related vascular remodeling and low-grade systemic inflammation may contribute to changes in aortic elastic properties and inflammatory biomarkers, particularly in symptomatic women. **Objective:** This study aimed to compare aortic elastic properties and albumin-based inflammatory indices between dyspneic and asymptomatic third-trimester pregnant women. A secondary aim was to establish reference values for echocardiographic and biomarker parameters in dyspneic pregnancy. **Methods:** In this prospective observational study, third-trimester pregnant women (≥27 gestational weeks) presenting to the emergency department (ED) with dyspnea were consecutively enrolled and compared with age-matched asymptomatic pregnant controls. Demographic, laboratory, and echocardiographic data were recorded. Aortic strain, aortic distensibility, and aortic stiffness were calculated using transthoracic echocardiography. Albumin-based inflammatory indices, including the hemoglobin–albumin–lymphocyte–platelet (HALP) score, prognostic nutritional index (PNI), C-reactive protein-to-albumin ratio (CAR), and RDW-to-albumin ratio (RAR), were analyzed. Receiver operating characteristic (ROC) and correlation analyses were performed. **Results:** A total of 241 pregnant women were included (121 dyspneic, 120 controls). Demographic characteristics were comparable between groups. Dyspneic pregnant women exhibited significantly lower aortic strain and aortic distensibility and higher aortic stiffness compared with controls (for all *p* < 0.05). Among laboratory parameters, CAR levels were significantly elevated in the dyspneic group (*p* < 0.001), whereas HALP, PNI, and RAR did not differ significantly. After adjustment for potential confounders, differences in aortic elastic properties remained significant. CAR demonstrated moderate discriminative ability for dyspnea (AUC = 0.692), while aortic elastic parameters showed modest predictive performance. In combined prediction models incorporating CAR with echocardiographic parameters, discriminatory performance improved, with area under the curve values exceeding 0.70. Weak positive correlations were observed between PNI and aortic strain and distensibility. **Conclusions:** Dyspneic third-trimester pregnant women exhibit impaired aortic elastic properties and increased CAR levels, suggesting the presence of subclinical vascular and inflammatory alterations. Assessment of aortic elasticity and CAR may provide a simple and practical approach for early cardiovascular risk stratification in symptomatic pregnancy, particularly in ED settings. Further multicenter studies with longitudinal follow-up are warranted to clarify their prognostic significance.

## 1. Introduction

Cardiovascular diseases are observed in approximately 1–4% of pregnant women worldwide and represent one of the leading causes of maternal morbidity and mortality [1]. Dyspnea is a common complaint during pregnancy and is often considered a benign physiological finding; however, it may also be an early manifestation of underlying cardiovascular pathology. Pregnancy-related hemodynamic changes, including increased blood volume, cardiac output, and heart rate, substantially increase cardiovascular demand and may unmask previously silent cardiac conditions [2]. Therefore, pregnant women presenting with dyspnea require careful clinical and echocardiographic evaluation to exclude subclinical cardiovascular disease [3].

Hormonal and physiological alterations during pregnancy lead to significant changes in vascular structure and function, particularly affecting large elastic arteries such as the aorta. These changes include dilation of the aorta and pulmonary arteries, accompanied by alterations in their elastic properties [4]. The elastic behavior of the aorta plays a crucial role in modulating left ventricular function and coronary perfusion. Impairment of aortic elasticity has been demonstrated in patients with coronary artery disease and is associated with adverse cardiovascular outcomes [5]. Aortic elastic properties can be noninvasively assessed using echocardiographic indices such as aortic strain, distensibility, compliance, and stiffness index [6]. Increased aortic stiffness reflects early vascular remodeling, vascular inflammation, and subclinical vascular sclerosis [7,8]. Previous studies have shown that aortic elastic properties are altered in women with a history of preeclampsia [9], and changes in aortic elasticity have also been reported in healthy pregnant women compared with nonpregnant controls [10].

Normal pregnancy is characterized by a mild systemic inflammatory response, which becomes more pronounced during the third trimester. To meet the metabolic and physiological demands of the developing fetus, pregnancy induces multiple biochemical and hematological alterations that may directly or indirectly influence inflammatory and nutritional biomarkers [11]. Serum albumin, a negative acute-phase reactant reflecting nutritional status and systemic inflammation, has gained increasing attention in pregnancy-related research. Recent studies have investigated albumin-based inflammatory and nutritional indices in various obstetric conditions. Lower albumin levels have been reported in women with recurrent pregnancy loss compared with healthy controls [12]. In patients with HELLP syndrome, albumin-based indices such as the hemoglobin–albumin–lymphocyte–platelet (HALP) score, prognostic nutritional index (PNI), and C-reactive protein-to-albumin ratio (CAR) were shown to differ significantly from those of normotensive pregnant women [13]. Similarly, reduced HALP scores have been observed in patients with preeclampsia [14], while both HALP and PNI have been proposed as early prognostic markers for fetal growth restriction when assessed during the first trimester [15]. Moreover, elevated CAR levels have been associated with disease severity and prognosis in pregnant patients with severe COVID-19 infection [16].

Despite accumulating evidence regarding vascular remodeling and albumin-based inflammatory indices in pregnancy, no previous study has simultaneously evaluated aortic elastic properties and albumin-based inflammatory indices in pregnant women presenting with dyspnea. Dyspnea during pregnancy is a multifactorial symptom and may arise from non-cardiovascular mechanisms such as physiological hyperventilation, altered respiratory mechanics, and reduced thoracic compliance. Nevertheless, pregnancy is also characterized by profound hemodynamic changes, increased arterial load, and vascular remodeling, which may lead to subtle impairments in arterial elasticity. Alterations in aortic elastic properties may contribute to increased cardiovascular load and symptom perception, even in the absence of overt cardiac disease, particularly in late pregnancy. Albumin-based inflammatory indices represent a heterogeneous group of biomarkers reflecting different aspects of nutritional status, inflammation, and immune response. While individual indices may demonstrate varying degrees of association with clinical phenotypes, their combined assessment allows a more comprehensive characterization of low-grade inflammatory burden in pregnancy. Therefore, the primary aim of this study was to investigate whether aortic elastic properties and albumin-based inflammatory indices differ between dyspneic and asymptomatic third-trimester pregnant women. The secondary aim was to establish reference values for echocardiographic and biomarker parameters in dyspneic pregnancy.

## 2. Materials and Methods

### 2.1. Study Design and Patient Population

This prospective, observational, single-center study was conducted between 1 April 2025 and 1 December 2025. Pregnant women in their third trimester (≥27 gestational weeks) who presented to the emergency department (ED) with a primary complaint of dyspnea were consecutively screened for eligibility. An age-matched control group was formed from asymptomatic third-trimester pregnant women without dyspnea who volunteered to participate during the same period. Asymptomatic controls were recruited from the emergency department but presented for non-inflammatory and non-cardiopulmonary reasons and had no history of dyspnea during the current pregnancy. None of the control participants reported dyspnea at presentation or during prior antenatal follow-up. Asymptomatic control participants were recruited from the emergency department for non-inflammatory and non-cardiopulmonary reasons, including routine obstetric evaluation, minor trauma without tissue injury, or administrative referral. None of the control participants presented with acute infectious symptoms, cardiopulmonary complaints, or conditions known to influence systemic inflammatory markers. Patients with any clinical or laboratory evidence of acute inflammation were systematically excluded to minimize selection bias. Although the study was prospectively designed, all clinical, laboratory, and echocardiographic assessments were performed at the time of emergency department presentation, and no longitudinal maternal or fetal outcome follow-up was planned. Therefore, the analyses reflect cross-sectional associations within a prospectively enrolled cohort.

Demographic characteristics, laboratory parameters, and echocardiographic findings were recorded and compared between the dyspnea and control groups. Recorded demographic characteristics included age, gestational week, parity, body mass index, systolic and diastolic blood pressure, and obstetric history. Laboratory parameters comprised hemoglobin, albumin, C-reactive protein, red cell distribution width, platelet count, lymphocyte count, and derived albumin-based inflammatory indices, including the hemoglobin–albumin–lymphocyte–platelet (HALP) score, prognostic nutritional index (PNI), C-reactive protein-to-albumin ratio (CAR), and RDW-to-albumin ratio (RAR). The study protocol was approved by the Ethics Committee of Necmettin Erbakan University Faculty of Medicine (approval date: 21 March 2025; approval number: 2025/5664 [23850.R1]). Written informed consent was obtained from all participants prior to enrollment. The study was conducted in accordance with the principles of the Declaration of Helsinki.

### 2.2. Inclusion and Exclusion Criteria

Pregnant women aged ≥18 years with complete hospital records and available laboratory and echocardiographic data were eligible for inclusion. Exclusion criteria included: age <18 years, obesity, gestational age ≤27 weeks, multiple pregnancy, history of diabetes mellitus, asthma, chronic obstructive pulmonary disease, hypertension, acute or chronic hematological disorders, liver or kidney disease, malignancy, acute or chronic cardiac or valvular disease, pulmonary embolism, preeclampsia or eclampsia, immunosuppressive conditions, active pulmonary infection, recent trauma, refusal to provide informed consent, or incomplete medical records. Only patients meeting all inclusion criteria and none of the exclusion criteria were included in the final analysis.

### 2.3. Clinical and Laboratory Data Collection

At the time of ED admission, the following parameters were recorded using a standardized data collection form: age, body mass index (BMI), gravidity, gestational week, mean arterial pressure (MAP), pulse pressure, white blood cell count (WBC), hemoglobin, platelet count, lymphocyte count, monocyte count, neutrophil count, red blood cell distribution width (RDW-CV), troponin I, C-reactive protein (CRP), serum albumin, thyroid-stimulating hormone (TSH), glucose, creatinine, and triglyceride levels. Echocardiographic and Doppler measurements were interpreted and recorded by a cardiologist blinded to the clinical group allocation.

### 2.4. Echocardiographic Examination

All transthoracic echocardiographic examinations were performed by the same experienced cardiologist using a standardized protocol. All aortic diameter measurements were obtained from the ascending aorta at approximately 3 cm above the aortic valve, using the parasternal long-axis view, in accordance with standard echocardiographic recommendations. Parasternal long-axis, short-axis, apical four-chamber, and subcostal views were routinely obtained in accordance with the American Society of Echocardiography (ASE) guidelines [17]. Left and right ventricular dimensions, chamber sizes, systolic and diastolic function parameters, and Doppler measurements were recorded. Aortic elastic properties were calculated using echocardiographic measurements of the ascending aorta, as previously described [7]. The following indices were derived:

Aortic strain (%) = (AoSD − AoDD) × 100/AoDD Aortic stiffness index (β) = ln(SBP/DBP)/[(AoSD − AoDD)/AoDD] Aortic distensibility (cm^2^/dyn/10^3^) = 2 × [100 × (AoSD − AoDD)/AoDD]/(SBP − DBP) (1)

where AoSD and AoDD represent systolic and diastolic aortic diameters, SBP is systolic blood pressure, DBP is diastolic blood pressure, and ln denotes the natural logarithm.

### 2.5. Albumin-Based Inflammatory and Nutritional Indices

The following albumin-based indices were calculated:

HALP score = hemoglobin (g/dL) × albumin (g/L) × lymphocyte count (10^3^/µL)/platelet count (10^9^/L), Prognostic Nutritional Index (PNI) = 10 × albumin (g/L) + 0.005 × total lymphocyte count (10^3^/µL), C-reactive protein-to-albumin ratio (CAR) = CRP (mg/L)/albumin (g/L), RDW-to-albumin ratio (RAR) = RDW (%)/albumin (g/L),(2)

The selection of HALP, PNI, CAR, and RAR was exploratory, based on their emerging role as inflammatory and nutritional biomarkers in pregnancy.

### 2.6. Hematological and Biochemical Analysis

Complete blood count parameters were measured using an automated hematology analyzer (Mindray BC-6800, Shenzhen, China). Biochemical analyses were performed with an automated chemistry analyzer (Mindray BS-2000M, Shenzhen, China). Transthoracic echocardiographic imaging was conducted using a Philips EPIQ CVx ultrasound system (Philips Healthcare, Andover, MA, USA).

### 2.7. Statistical Analysis

Statistical analyses were performed using SPSS version 30.0 (IBM Corp., Chicago, IL, USA). The normality of continuous variables was assessed using the Kolmogorov–Smirnov test, histogram analysis, skewness–kurtosis values, and Q–Q plots. Continuous variables were expressed as mean ± standard deviation or median (interquartile range), as appropriate, while categorical variables were presented as frequency and percentage.

Between-group comparisons were performed using the independent samples *t*-test or Mann–Whitney U test for continuous variables, and Fisher’s exact test for categorical variables. Homogeneity of variances was evaluated using Levene’s test. Potential confounding variables were controlled using analysis of covariance (ANCOVA). ANCOVA was preferred over ANOVA to allow adjustment for potential confounding variables, including age, gestational week, and body mass index, which may influence vascular and inflammatory parameters. Receiver operating characteristic (ROC) curve analysis was conducted to determine the cut-off values and predictive performance of selected variables. Correlations between continuous variables were assessed using Pearson or Spearman correlation analysis, depending on data distribution. A two-sided *p* value <0.05 was considered statistically significant, with a 95% confidence interval.

## 3. Results

### 3.1. Patient Characteristics and Laboratory Findings

**Figure 1:**
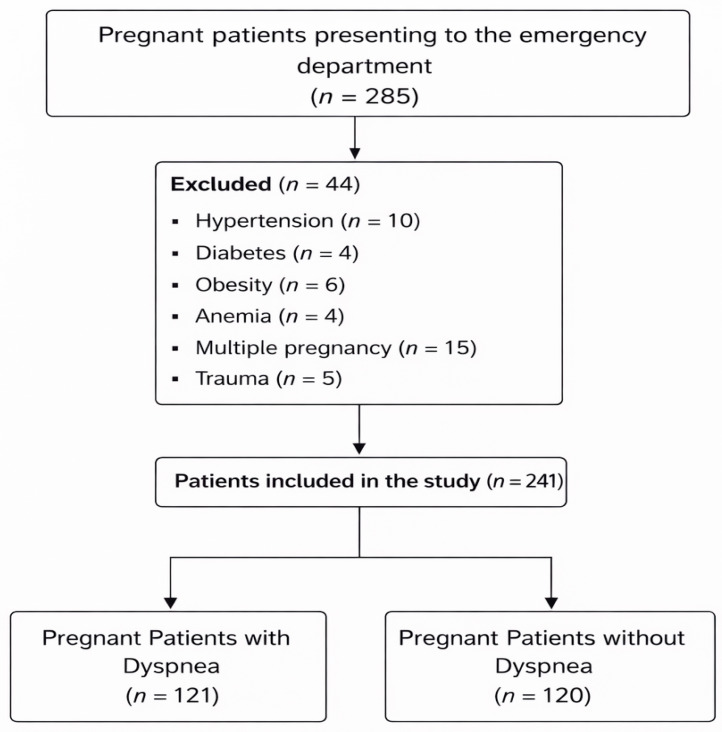
Patient flow diagram showing enrollment, exclusions, and group allocation.

A total of 241 third-trimester pregnant women were included in the final analysis, comprising 120 asymptomatic controls (49.8%) and 121 dyspneic patients (50.2%). The patient flow diagram illustrating enrollment, exclusions, and group allocation is presented in Figure 1.

**Figure 2:**
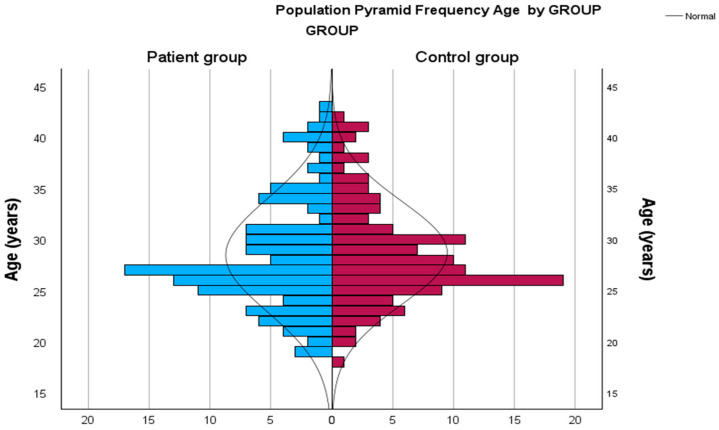
Distribution of age (years) among groups (*p* = 0.729). Blue bars represent the patient group and red bars represent the control group.

Baseline demographic and laboratory characteristics of the study population are summarized in Table 1. There were no significant differences between the dyspnea and control groups with respect to age, body mass index (BMI), gravidity, gestational week, mean arterial pressure (MAP), or pulse pressure (for all *p* > 0.05). The age distribution between groups is illustrated in Figure 2.

When laboratory parameters were compared, no significant differences were observed between groups in terms of glucose, creatinine, TSH, troponin I, albumin, hemoglobin, platelet count, lymphocyte count, monocyte count, RDW-CV, HALP score, PNI, or RAR (for all *p* > 0.05). In contrast, triglyceride levels, CRP, WBC, neutrophil count, and CAR were significantly higher in the dyspneic group compared with controls (for all *p* < 0.001). Although statistically significant, the absolute differences in inflammatory markers between groups were modest and should be interpreted cautiously.

### 3.2. Echocardiographic Findings

Comparisons of echocardiographic parameters between the dyspnea and control groups are presented in Table 2. No significant differences were observed between groups in left ventricular end-diastolic diameter (LVEDd), left ventricular end-systolic diameter (LVESd), interventricular septum thickness (IVSd), left ventricular posterior wall thickness (LVPWd), left atrial diameter, aortic root diameter, aortic systolic diameter, aortic diastolic diameter, tricuspid annular plane systolic excursion (TAPSE), mitral E and A wave velocities, septal A′ and lateral E′ and A′ wave velocities, aortic systolic diameter index, aortic diastolic diameter index, or degree of tricuspid regurgitation (for all *p* > 0.05).

However, the dyspneic group demonstrated significantly higher right atrial diameter, right ventricular diameter, systolic pulmonary artery pressure (sPAP), septal E/e′, lateral E/e′, and aortic stiffness compared with the control group (for all *p* < 0.05). In contrast, septal E′ wave velocity, S′ velocity, aortic strain, and aortic distensibility were significantly lower in dyspneic patients (for all *p* < 0.05).

### 3.3. Adjustment for Confounding Factors

To account for potential confounding effects, comparisons of aortic elastic properties were adjusted for age, BMI, MAP, and gestational week using ANCOVA. After adjustment, the observed differences in aortic strain, aortic distensibility, and aortic stiffness between the dyspnea and control groups remained statistically significant, indicating that these associations were independent of the selected covariates (Table 3).

### 3.4. ROC Analysis

ROC analyses were performed to assess the discriminative performance of laboratory and echocardiographic parameters in identifying dyspneic pregnancy (Table 4). Among laboratory parameters, CAR demonstrated a cut-off value of ≥2.34, with 47.1% sensitivity, 90.8% specificity, and an area under the curve (AUC) of 0.692 (*p* < 0.001).

**Figure 3:**
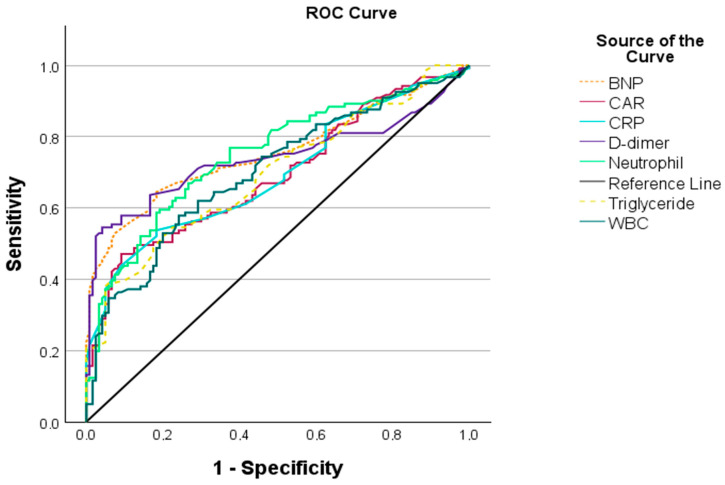
Receiver operating characteristic (ROC) curves of laboratory parameters for distinguishing dyspneic pregnant women from asymptomatic controls.

**Figure 4:**
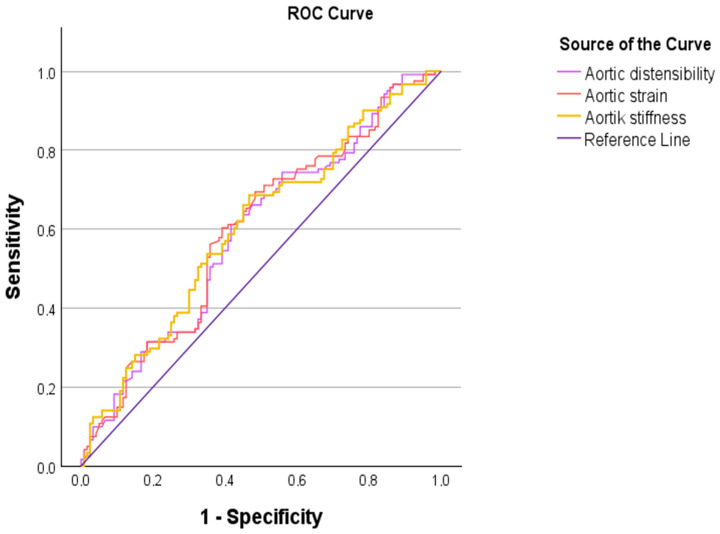
Receiver operating characteristic (ROC) curves of echocardiographic parameters for distinguishing dyspneic pregnant women from asymptomatic controls.

**Figure 5:**
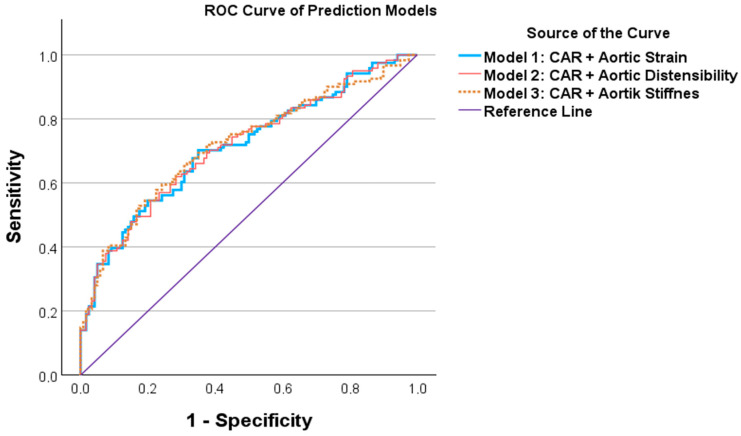
The ROC analyses of prediction models in terms of predicting patient group. Receiver operating characteristic curves demonstrating the discriminatory performance of combined models incorporating CAR with echocardiographic parameters. All combined models achieved AUC values ≥ 0.70, indicating acceptable discriminatory ability.

Among echocardiographic parameters, an aortic strain cut-off value of ≤0.338 yielded 60.3% sensitivity, 60.8% specificity, and an AUC of 0.598 (*p* < 0.05). An aortic distensibility cut-off value of ≤0.017 demonstrated 61.2% sensitivity, 58.3% specificity, and an AUC of 0.592 (*p* < 0.05). An aortic stiffness cut-off value of ≥1.325 showed 68.6% sensitivity, 53.3% specificity, and an AUC of 0.604 (*p* < 0.05). ROC curves for laboratory and echocardiographic parameters are illustrated in Figure 3 and Figure 4, respectively. The ROC analysis of the combined prediction models is presented in Figure 5.

### 3.5. Correlation Analysis

Correlation analyses between age, BMI, MAP, albumin-based indices, and echocardiographic parameters are presented in Table 5. PNI demonstrated a weak but statistically significant positive correlation with aortic strain (rho = 0.165, *p* < 0.05) and aortic distensibility (rho = 0.158, *p* < 0.05).

Age showed a moderate negative correlation with aortic strain (rho = −0.730, *p* < 0.001) and aortic distensibility (rho = −0.721, *p* < 0.001), and a moderate positive correlation with aortic stiffness (rho = 0.602, *p* < 0.001). BMI was weakly correlated with aortic strain (rho = −0.273, *p* < 0.001), aortic distensibility (rho = −0.274, *p* < 0.001), and aortic stiffness (rho = 0.245, *p* < 0.001). MAP demonstrated moderate correlations with aortic strain (rho = −0.697, *p* < 0.001), aortic distensibility (rho = −0.709, *p* < 0.001), and aortic stiffness (rho = 0.499, *p* < 0.001).

### 3.6. Subgroup Analysis According to Parity

Subgroup analysis was performed to evaluate the potential impact of parity on aortic elastic properties and inflammatory indices within the dyspneic group. No statistically significant differences were observed between primigravid and multigravid women in terms of aortic strain, aortic distensibility, aortic stiffness, or CAR levels (all *p* > 0.05).

### 3.7. Multivariable Logistic Regression Analysis

In multivariable logistic regression analysis, CAR ≥ 2.34 and aortic stiffness ≥ 1.325 remained independently associated with dyspnea after adjustment for age, body mass index, and gestational week (Table 6). Other covariates did not show independent associations.

## 4. Discussion

Pregnancy is a dynamic physiological state characterized by profound hemodynamic and cardiovascular adaptations. These changes, while generally well tolerated, may unmask previously silent cardiovascular abnormalities, particularly in women presenting with symptoms such as dyspnea. Comprehensive clinical assessment, risk stratification, and appropriate imaging modalities constitute the cornerstone of managing pregnant women with suspected cardiovascular involvement [18]. In the present study, we evaluated aortic elastic properties and albumin-based inflammatory indices in dyspneic third-trimester pregnant women and demonstrated significant alterations in aortic stiffness, strain, and distensibility, along with elevated CAR levels, compared with asymptomatic controls.

Hormonal and structural vascular changes during pregnancy adversely affect elastic fiber composition and promote hypertrophy of aortic smooth muscle cells, leading to progressive dilation of the aorta and increased wall stress. These changes may persist into the postpartum period and, in some cases, may not fully regress [4]. Arterial stiffness is strongly influenced by systolic blood pressure, whereas arterial distensibility more directly reflects intrinsic mechanical properties of large arteries. Consequently, increased arterial stiffness and reduced distensibility impair left ventricular–arterial coupling and increase myocardial oxygen demand [19]. Previous studies have demonstrated a close relationship between arterial stiffness and inflammation in healthy individuals, hypertensive patients, and those with chronic inflammatory disorders [7,8,20].

Echocardiographic assessment of aortic elastic properties provides a noninvasive and practical method for evaluating vascular remodeling. Increased aortic stiffness and reduced aortic strain and distensibility are considered markers of early vascular dysfunction [7,19]. Pathological aortic stiffness has been associated with multiple cardiovascular and systemic conditions, including hypertension, diabetes mellitus, obesity, aging, subclinical inflammation, and atherosclerosis [4,7,19,21,22]. In pregnancy-specific contexts, impaired aortic elasticity has been linked to the development and severity of preeclampsia [9,23], and increased parity and advancing gestational age have been shown to further exacerbate arterial stiffness [4,24]. Ulusoy et al. reported higher aortic diameter, distensibility, and strain values with lower aortic stiffness indices in healthy pregnant women compared with nonpregnant controls, attributing these findings to elevated estrogen levels and a hyperdynamic circulatory state [10]. In contrast, Orabona et al. suggested that pregnancy-related impairment of aortic elastic properties may increase vulnerability to aortic complications, including dissection, particularly in high-risk populations [9]. More recently, Turi et al. emphasized that increased arterial stiffness during pregnancy may be associated with a broad spectrum of fetomaternal pathologies [25]. Consistent with these observations, our study demonstrated significantly lower aortic strain and distensibility and higher aortic stiffness in dyspneic pregnant women compared with asymptomatic controls. Although these parameters showed statistically significant differences, their discriminatory power was modest, as reflected by relatively low AUC values. Nevertheless, these findings suggest that dyspnea in pregnancy may be associated with subclinical vascular dysfunction rather than overt structural heart disease. The relatively large number of third-trimester pregnant women presenting with dyspnea reflects the high clinical relevance of this symptom in emergency department settings, where dyspnea often prompts evaluation to exclude cardiopulmonary or obstetric complications. Importantly, subgroup analysis revealed that parity did not significantly influence aortic elastic parameters or inflammatory indices within the dyspneic group, suggesting that the observed associations were not primarily driven by differences in pregnancy-related cardiovascular adaptation between primigravid and multigravid women. Dyspnea represents the primary clinical phenotype of the present study and should be interpreted as a multifactorial symptom rather than a direct manifestation of overt cardiovascular disease. In this context, the observed impairment in aortic elastic properties and the presence of low-grade inflammatory activity may contribute to increased cardiovascular load and heightened symptom perception during late pregnancy. These subclinical vascular alterations may lower the physiological threshold for dyspnea, even in the absence of structural heart disease or overt hemodynamic compromise.

Pregnancy is also characterized by adaptive changes in the immune system, balancing immune tolerance toward the fetus with controlled inflammatory activation [26]. Maternal immune responses to fetal and trophoblastic alloantigens may result in low-grade systemic inflammation [27]. While physiological inflammation and oxidative stress are essential components of normal pregnancy [28], excessive or dysregulated inflammatory responses have been associated with adverse maternal and neonatal outcomes [29,30]. Serum albumin, a negative acute-phase reactant synthesized by the liver, reflects both nutritional status and systemic inflammation and has emerged as a clinically relevant biomarker in pregnancy-related conditions [13]. Several studies have demonstrated associations between hypoalbuminemia and increased disease severity or adverse maternal outcomes, particularly in hypertensive disorders of pregnancy and HELLP syndrome [31,32]. Albumin-based indices, such as the HALP score, PNI, and CAR, integrate hematological, inflammatory, and nutritional parameters and have gained increasing attention in obstetric research. HALP has been proposed as a useful predictor of hyperemesis gravidarum severity and preterm birth risk [33,34], although conflicting results have also been reported, particularly in early pregnancy and preeclampsia cohorts [35]. In our study, HALP scores did not differ significantly between dyspneic and asymptomatic pregnant women, nor did they correlate with aortic elastic properties. This finding may be attributable to the inclusion of otherwise healthy pregnant women without overt obstetric or systemic pathology. It is important to emphasize that CRP is a non-specific marker of systemic inflammation and was not interpreted as a dyspnea-specific biomarker in the present study. To minimize confounding, patients with overt infection or acute inflammatory conditions were excluded. Accordingly, CRP and CAR were evaluated as indicators of low-grade inflammatory burden that may coexist with dyspnea during late pregnancy, rather than as direct causal determinants of the symptom. It should be noted that although CRP and CAR levels were significantly higher in the dyspneic group, the absolute magnitude of these differences was relatively small. Given that both groups were recruited from the emergency department, residual confounding from unmeasured acute or subclinical conditions cannot be completely excluded, and therefore, inflammatory findings should be interpreted cautiously. Among the albumin-based inflammatory indices evaluated, CAR demonstrated the most pronounced difference between dyspneic and asymptomatic pregnant women. Nevertheless, the study was designed to assess a panel of albumin-based indices rather than a single biomarker, as the absence of significant changes in other indices provides important contextual information and underscores the heterogeneous nature of inflammatory responses during pregnancy.

PNI, a composite marker of nutritional and immunological status, has been increasingly investigated in pregnancy-related disorders [36,37]. Reduced PNI levels have been associated with early-onset preeclampsia and adverse cardiovascular outcomes in peripartum cardiomyopathy [37,38]. In the present study, PNI values were comparable between groups; however, weak positive correlations were observed between PNI and both aortic strain and distensibility. These findings suggest a potential link between maternal nutritional–inflammatory status and vascular elasticity, although the clinical significance of this association appears limited.

CAR has emerged as a robust marker of systemic inflammation in various pregnancy-related conditions. Elevated CAR levels have been associated with disease severity in pregnant patients with COVID-19 [16], preterm premature rupture of membranes, and increased thrombotic risk [39,40]. In our cohort, CAR levels were significantly higher in dyspneic pregnant women and demonstrated moderate discriminative ability for dyspnea, with high specificity but limited sensitivity. These results indicate that CAR may serve as a complementary biomarker reflecting inflammatory burden in symptomatic pregnancy.

Hematological parameters undergo substantial physiological alterations during pregnancy and have increasingly been investigated as potential predictors of adverse maternal and fetal outcomes [41]. Recent studies have highlighted the prognostic value of inflammation-based hematological indices in obstetric populations, particularly for fetal growth restriction and adverse neonatal outcomes [42,43,44,45,46,47,48]. Red blood cell distribution width (RDW), a marker reflecting systemic inflammation and oxidative stress, has been associated with various pregnancy-related complications, including preeclampsia, intrahepatic cholestasis of pregnancy, acute pancreatitis, and recurrent pregnancy loss [43,44,45,46,47,48]. RDW and RDW-based ratios, such as RAR, have been proposed as markers of systemic inflammation and disease severity in several inflammatory and metabolic conditions [49,50,51,52,53]. Despite this, our study did not identify significant differences in RDW, albumin, or RAR between dyspneic and asymptomatic pregnant women. Although these findings contrast with some reports in pathological pregnancy states, they may provide valuable reference data for healthy third-trimester populations and underscore the need for further investigation. From a clinical perspective, the present findings suggest that assessment of aortic elastic properties and albumin-based inflammatory indices, particularly CAR, may provide complementary information in the evaluation of dyspneic pregnant women presenting to the emergency department. Although these parameters are not intended for diagnostic decision-making, they may help identify subclinical vascular and inflammatory burden in otherwise healthy pregnant women. This approach may contribute to early risk stratification, closer monitoring, and hypothesis generation for future longitudinal studies designed to evaluate maternal and fetal outcomes.

Although the combined models achieved AUC values exceeding 0.70, these results indicate moderate discriminatory performance and should not be interpreted as supporting immediate clinical implementation. Instead, they suggest the presence of a measurable vascular–inflammatory association in dyspneic pregnancy that requires validation in larger, longitudinal cohorts.

## 5. Limitations

Several limitations of this study should be acknowledged. First, this was a single-center study with a relatively limited sample size, which may restrict the generalizability of the findings to broader and more diverse populations. Accordingly, the findings should be interpreted as cross-sectional associations observed within a prospectively enrolled cohort, rather than as longitudinal or causal relationships. Nevertheless, the comparable demographic and clinical characteristics of the dyspneic and control groups support the internal validity of the study. Second, owing to its prospective observational design, the identified associations should be interpreted as correlational rather than causal. In addition, feto-maternal and long-term clinical outcomes were not systematically followed, precluding evaluation of the clinical implications of the observed alterations in aortic elastic properties and CAR levels. Third, although all echocardiographic examinations were performed by an experienced cardiologist using a standardized protocol, intra-observer variability was not formally assessed, which may limit the reproducibility of certain measurements. Furthermore, aortic elastic properties were evaluated solely using transthoracic echocardiography and were not validated with advanced imaging modalities such as cardiac magnetic resonance imaging. Fourth, to minimize physiological variability, only third-trimester pregnant women were included in the study. While this approach enhanced group homogeneity, it prevented assessment of hemodynamic and inflammatory changes across different trimesters. Additionally, postpartum follow-up data were unavailable, making it unclear whether the observed changes represent transient or persistent vascular alterations. Hormonal parameters, particularly progesterone levels, which may influence respiratory physiology during pregnancy, were not assessed in this study. Hormonal profiling was beyond the scope of the present investigation and is not routinely performed in emergency department settings; however, future studies incorporating hormonal measurements may provide additional insight into the multifactorial mechanisms underlying dyspnea during pregnancy. Iron parameters were not evaluated in the present study. Although iron deficiency may contribute to dyspnea during pregnancy, patients with clinically significant anemia were excluded, and hemoglobin levels were assessed as part of routine laboratory evaluation. Detailed iron studies were not routinely available in the emergency department setting and should be considered in future studies. No standardized questionnaire was used to assess dyspnea severity or symptom burden in this study. Dyspnea was evaluated clinically at the time of emergency department presentation, in accordance with routine practice. The absence of validated symptom scoring represents a limitation, and future studies incorporating standardized questionnaires may provide a more comprehensive assessment of symptom severity and its relationship with vascular and inflammatory parameters. Lifestyle characteristics and occupational factors, which may influence physical activity levels and symptom perception, were not systematically assessed in this study. Given the emergency department setting and the acute nature of presentation, detailed evaluation of lifestyle or occupational exposures was not feasible. Future studies may incorporate structured assessments to evaluate the potential impact of these factors on dyspnea during pregnancy. Environmental and residential factors, including air pollution exposure, were not specifically assessed in this study. As participants were recruited from a single tertiary center serving a relatively homogeneous urban population, detailed evaluation of environmental exposures was beyond the scope of the present investigation. Future multicenter studies incorporating environmental and regional data may help clarify the influence of such factors on dyspnea during pregnancy. Finally, dyspnea is a heterogeneous and subjective symptom, particularly in ED settings. The severity, duration, and temporal characteristics of dyspnea were not quantified using a standardized scoring system. Moreover, despite the exclusion of major cardiopulmonary and systemic conditions and statistical adjustment for potential confounders using ANCOVA, residual confounding due to unmeasured pulmonary, metabolic, or psychosocial factors cannot be entirely excluded. Despite these limitations, this study represents one of the few investigations to simultaneously evaluate aortic elastic properties and albumin-based inflammatory indices in dyspneic third-trimester pregnant women. The findings should therefore be considered hypothesis-generating and may serve as a reference framework for future large-scale, multicenter studies with longitudinal follow-up. The limitations of the present study were deliberately described in detail to ensure transparency and to avoid overinterpretation of the findings. These limitations also serve to define a clear framework for future research. Prospective multicenter studies with longitudinal maternal and fetal outcome follow-up, incorporation of hormonal and iron parameters, validated symptom questionnaires, and assessment of lifestyle and environmental factors are warranted to further elucidate the mechanisms underlying dyspnea during pregnancy and to determine the prognostic significance of vascular and inflammatory alterations.

## 6. Conclusions

In this prospective observational study, dyspneic third-trimester pregnant women exhibited impaired aortic elastic properties and elevated CAR levels compared with asymptomatic controls, suggesting the presence of subclinical vascular and inflammatory alterations. Although the discriminatory performance of individual parameters was moderate, multivariable and combined model analyses indicate a measurable biological signal linking dyspnea with vascular–inflammatory interplay in late pregnancy.

These findings should be interpreted as hypothesis-generating, rather than as evidence supporting immediate clinical implementation. The results provide pathophysiological insight into symptomatic pregnancy and may serve as a foundation for future multicenter, longitudinal studies aimed at clarifying the prognostic significance of vascular and inflammatory markers in this population.

## Figures and Tables

**Table 1:** Comparison of demographic and laboratory parameters of patient and control groups.

Variables	Control Group (*n* = 120, 49.8%)	Patient Group (*n* = 121, 50.2%)	Overall	*p*-Value
Age, years	28.68 ± 5.05	28.45 ± 5.55	28.56 ± 5.3	0.729 ^a^
BMI, kg/m^2^	29.33 (20.31–44.63)	29.3 (20.08–45.79)	29.3 (20.08–45.79)	0.965 ^b^
Gravidity	2 (1–7)	2 (1–7)	2 (1–7)	0.054 ^b^
Gestational week	33.16 ± 3.92	33.12 ± 3.88	33.14 ± 3.89	0.932 ^a^
MAP	84.65 ± 5.19	84.53 ± 5.36	84.59 ± 5.27	0.865 ^a^
Pulse pressure	40.4 ± 1.3	40.4 ± 1.3	40.4 ± 1.3	0.860 ^a^
Laboratory parameters				
Glucose (mg/dL)	87 (60–122)	88 (57–139)	87 (57–139)	0.228 ^b^
Creatinine (mg/dL)	0.5 (0.16–0.81)	0.5 (0.16–0.81)	0.5 (0.16–0.81)	0.975 ^b^
TSH (µUI/mL)	1.88 (0.31–5.52)	1.89 (0.31–5.52)	1.89 (0.31–5.52)	0.705 ^b^
Troponin I (ng/L)	2.5 (2.5–6)	2.5 (2.5–17)	2.5 (2.5–17)	0.869 ^b^
Triglyceride (mg/dL)	152.5 (114–180)	165 (128–246)	156 (114–246)	<0.001 ^b^
CRP (mg/L)	5 (2–13)	8 (1.88–37)	6 (1.88–37)	<0.001 ^b^
Albumin (g/L)	35.8 ± 2.4	35.8 ± 2.4	35.8 ± 2.4	0.872 ^a^
WBC (10^9^/L)	8.16 (5.16–13.72)	9.46 (5.16–17.72)	8.62 (5.16–17.72)	<0.001 ^b^
Hb (g/dL)	11.8 (9.9–15)	11.8 (9.9–15)	11.8 (9.9–15)	0.809 ^b^
PLT (10^9^/L)	240 (115–503)	240 (115–503)	240 (115–503)	0.872 ^b^
Lymphocyte (10^3^/µL)	1.8 (1.03–3.44)	1.79 (1.03–3.44)	1.79 (1.03–3.44)	0.943 ^b^
Monocyte (%)	0.56 (0.29–1.06)	0.55 (0.29–1.06)	0.56 (0.29–1.06)	0.885 ^b^
Neutrophil (10^9^/L)	5.58 (3.21–9.7)	6.92 (3.21–13.7)	6.08 (3.21–13.7)	<0.001 ^b^
RDW-CV(%)	14.1 (11.8–26.9)	14.1 (11.8–26.9)	14.1 (11.8–26.9)	0.990 ^b^
HALP	3.03 (1.24–7.95)	2.99 (1.24–7.95)	2.99 (1.24–7.95)	0.746 ^b^
CAR	1.3 (0.48–3.95)	2.13 (0.47–12.13)	1.59 (0.47–12.13)	<0.001 ^b^
PNI	35.85 ± 2.35	35.8 ± 2.36	35.83 ± 2.35	0.871 ^a^
RAR (%/g/dL)	0.4 (0.3–0.68)	0.4 (0.3–0.68)	0.4 (0.3–0.68)	0.964 ^b^

BMI body mass index, MAP mean arterial pressure, TSH thyroid-stimulating hormone, CRP C-reactive protein, WBC white blood cell, Hb hemoglobin, PLT platelet, RDW red blood cell distribution width, HALP Hemoglobin-Albumin-Lymphocyte-Platelet Score, CAR C-Reactive Protein-to-Albumin Ratio, PNI prognostic nutritional index, RAR RDW/albumin ratio. ^a^ Independent *t*-test, ^b^ Mann–Whitney U test.

**Table 2:** Comparison of echocardiography findings of patient and control groups.

Variables	Control Group (*n* = 120, 49.8%)	Patient Group (*n* = 121, 50.2%)	Overall	*p*-Value
LVEDd (mm)	44 (38–48)	44 (38–48)	44 (38–48)	0.734 ^b^
LVESd (mm)	27 (23–30)	27 (23–30)	27 (23–30)	0.613 ^b^
IVSd (mm)	10 (9–19)	10 (9–19)	10 (9–19)	0.690 ^b^
LVPWd (mm)	9 (8–10)	9 (8–10)	9 (8–10)	0.690 ^b^
Left atrium diameter (mm)	28 (23–36)	28 (23–36)	28 (23–36)	0.782 ^b^
Right atrium diameter (mm)	23.5 ± 1.6	24.1 ± 2	23.8 ± 1.9	0.011^a^
Right ventricle diameter (mm)	22.5 ± 1.6	23.1 ± 1.9	22.8 ± 1.8	0.005 ^a^
Aortic root diameter (mm)	24 (19–30)	24 (19–30)	24 (19–30)	0.796 ^b^
Aortic systolic diameter (mm)	24.5 (19.5–30.2)	24.5 (19.5–30.2)	24.5 (19.5–30.2)	0.796 ^b^
Aortic diastolic diameter (mm)	18.1 (11.9–28.2)	18.5 (12.4–28.5)	18.3 (11.9–28.5)	0.183 ^b^
sPAP (mmHg)	15 (5–25)	19 (5–32)	17 (5–32)	<0.001
TAPSE (mm)	25.6 ± 1.3	25.3 ± 1.3	25.5 ± 1.3	0.141 ^a^
Mitral E wave velocity (m/s)	81.5 ± 10.1	81.1 ± 10.5	81.3 ± 10.3	0.782 ^a^
Mitral A wave velocity (m/s)	60.1 ± 9.4	60.2 ± 9.5	60.1 ± 9.4	0.952 ^a^
Septal E′ wave velocity (m/s)	105 (96–143)	102 (63–130)	103 (63–143)	0.009 ^b^
Septal A′ wave velocity (m/s)	76.5 (56–91.1)	78 (45–91.1)	78 (45–91.1)	0.972 ^b^
Lateral E′ wave velocity (m/s)	151 (132–176)	151 (75–176)	151 (75–176)	0.211 ^b^
Lateral A′ wave velocity (m/s)	116 (78–137)	115 (40–168)	115 (40–168)	0.247 ^b^
Septal E/e′	0.74 (0.54–1.08)	0.76 (0.54–1.29)	0.75 (0.54–1.29)	0.049 ^b^
Lateral E/e′	0.53 (0.38–0.8)	0.55 (0.4–0.91)	0.54 (0.38–0.91)	0.018 ^b^
S′ (cm/s)	67 ± 1.2	66.2 ± 1.3	66.6 ± 1.3	<0.001 ^a^
Aortic systolic diameter index	12.97 ± 1.42	13 ± 1.48	12.99 ± 1.45	0.874 ^a^
Aortic diastolic diameter index	9.67 ± 1.29	9.86 ± 1.33	9.76 ± 1.31	0.259 ^a^
Tricuspid regurgitation				
*1*	0 (0%)	4 (3.3%)	4 (1.7%)	0.133 ^c^
*Minimal*	54 (45.0%)	57 (47.1%)	111 (46.1%)	
*Mild*	66 (55.0%)	60 (49.6%)	126 (52.3%)	
Aortic strain	0.35 (0.14–0.64)	0.33 (0.15–0.57)	0.34 (0.14–0.64)	0.008 ^b^
Aortic distensibility	0.017 ± 0.004	0.016 ± 0.004	0.017 ± 0.004	0.013 ^a^
Aortic stiffness	1.31 (0.8–3.22)	1.42 (0.89–3.02)	1.36 (0.8–3.22)	0.005 ^b^

LVEDd left ventricular end-diastolic diameter, LVESd left ventricle end-systolic diameter, IVSd interventricular septum thickness, LVPWd left ventricle posterior wall thickness, sPAP systolic pulmonary artery pressure, TAPSE tricuspid annular plane systolic excursion. ^a^ Independent *t*-test, ^b^ Mann–Whitney U test, ^c^ Fisher’s exact test.

**Table 3:** Adjustment for possible confounding factors (covariates) when comparing the elastic properties of the aorta between groups.

	Group			ANCOVA ^†^		
	Control Group (*n* = 120, 49.8%)	Patient Group (*n* = 121, 50.2%)				
	Distribution					
			*p*	*F*	*p*	*η_p_* ^2^
Aortic strain *	0.35 (0.14–0.64)	0.33 (0.15–0.57)	**0.008** ^b^	15.326	**<0.001**	0.061
Aortic distensibility	0.017 ± 0.004	0.016 ± 0.004	**0.013** ^a^	16.520	**<0.001**	0.066
Aortic stiffness *	1.31 (0.8–3.22)	1.42 (0.89–3.02)	**0.005** ^b^	17.087	**<0.001**	0.068

* Logarithmic transform has been performed in order to convert skewed variables into a normal distribution pattern. † Covariates used for adjustment in the model: Age, BMI, MAP and gestational week. ^a^ Independent *t*-test, ^b^ Mann–Whitney U test; significant values were expressed in bold.

**Table 4:** ROC analysis of variables in terms of dyspnea group.

	AUC	95% CI		Cut-Off ^†^	Sensitivity (%)	Specificity (%)	*p*
		Lower Limit	Upper Limit				
Triglyceride (mg/dL)	0.695	0.629	0.761	≥170.5	38.8%	94.2%	<0.001
CRP (mg/L)	0.694	0.627	0.760	≥7.15	53.7%	81.7%	<0.001
WBC (10^9^/L)	0.701	0.635	0.767	≥9.15	57.9%	74.2%	<0.001
Neutrophil	0.749	0.687	0.811	≥6.64	58.7%	81.7%	<0.001
CAR	0.692	0.625	0.758	≥2.34	47.1%	90.8%	<0.001
Aortic strain *	0.598	0.527	0.670	≤0.338	60.3%	60.8%	0.007
Aortic distensibility *	0.592	0.521	0.664	≤0.017	61.2%	58.3%	0.012
Aortic stiffness	0.604	0.532	0.675	≥1.325	68.6%	53.3%	0.004

Abbreviations: ROC = Receiver Operating Characteristic, AUC = area under the curve, CI = Confidence Interval. Reference category = Control group. † Cut-off values determined based on Youden J index. * Lower values are associated with positive (dyspnea) results.

**Table 5:** Correlation relationship between age, BMI, MAP, HALP, CAR, PNI, RAR, and echocardiography parameters.

		Age *	BMI	MAP *	HALP	CAR	PNI *	RAR
LVEDd (mm)	r/rho	0.242	0.319	0.254	−0.188	0.079	−0.160	0.043
	*p*-value	<0.001	<0.001	<0.001	0.003	0.220	0.013	0.510
LVESd (mm)	r/rho	0.355	0.202	0.349	−0.139	−0.009	−0.057	−0.053
	*p*-value	<0.001	0.002	<0.001	0.030	0.884	0.375	0.414
IVSd (mm)	r/rho	0.298	0.380	0.251	−0.181	0.170	−0.174	0.015
	*p*-value	<0.001	<0.001	<0.001	0.005	0.008	0.007	0.818
LVPWd mm	r/rho	0.301	0.378	0.255	−0.199	0.162	−0.185	0.004
	*p*-value	<0.001	<0.001	<0.001	0.002	0.012	0.004	0.953
Left atrium diameter (mm)	r/rho	0.206	0.152	0.164	−0.006	0.007	0.024	−0.083
	*p*-value	0.001	0.018	0.011	0.926	0.919	0.706	0.198
Right atrium diameter (mm) *	r/rho	0.543	0.282	0.515	−0.111	−0.021	−0.190	−0.004
	*p*-value	<0.001	<0.001	<0.001	0.084	0.743	0.003	0.956
Right ventricle diameter (mm) *	r/rho	0.529	0.283	0.507	−0.097	−0.016	−0.195	−0.010
	*p*-value	<0.001	<0.001	<0.001	0.132	0.805	0.002	0.876
Aortic root diameter (mm)	r/rho	0.292	0.160	0.241	−0.080	−0.088	−0.035	0.004
	*p*-value	<0.001	0.013	<0.001	0.213	0.172	0.584	0.948
Aortic systolic diameter (mm)	r/rho	0.260	0.113	0.187	−0.083	−0.109	−0.024	−0.009
	*p*-value	<0.001	0.081	0.004	0.197	0.092	0.712	0.887
Aortic diastolic diameter (mm)	r/rho	0.530	0.218	0.480	−0.113	−0.053	−0.118	−0.002
	*p*-value	<0.001	0.001	<0.001	0.080	0.416	0.068	0.970
sPAP (mmHg)	r/rho	0.349	0.012	0.359	−0.081	0.177	−0.092	−0.012
	*p*-value	<0.001	0.859	<0.001	0.212	0.006	0.153	0.852
TAPSE (mm) *	r/rho	−0.567	−0.281	−0.535	−0.148	−0.225	0.047	−0.049
	*p*-value	<0.001	<0.001	<0.001	0.022	<0.001	0.464	0.448
Mitral E wave velocity (m/s) *	r/rho	−0.048	0.045	−0.068	−0.048	0.034	0.084	−0.072
	*p*-value	0.461	0.490	0.290	0.455	0.603	0.194	0.265
Mitral A wave velocity (m/s) *	r/rho	0.065	0.021	0.033	−0.108	−0.010	−0.048	0.012
	*p*-value	0.315	0.744	0.614	0.096	0.874	0.461	0.853
Septal E′ wave velocity (m/s)	r/rho	−0.167	−0.098	−0.158	−0.228	−0.014	−0.309	0.208
	*p*-value	0.009	0.129	0.014	<0.001	0.826	<0.001	0.001
Septal A′ wave velocity (m/s)	r/rho	0.033	−0.187	0.028	−0.302	0.095	−0.323	0.196
	*p*-value	0.611	0.004	0.662	<0.001	0.140	<0.001	0.002
Lateral E′ wave velocity (m/s)	r/rho	−0.225	−0.173	−0.184	−0.319	−0.036	−0.272	0.099
	*p*-value	<0.001	0.007	0.004	<0.001	0.577	<0.001	0.127
Lateral A′ wave velocity (m/s)	r/rho	−0.097	−0.163	−0.065	−0.322	0.125	−0.255	0.057
	*p*-value	0.132	0.011	0.313	<0.001	0.052	<0.001	0.381
Septal E/e′	r/rho	0.123	0.110	0.121	0.139	0.057	0.272	−0.204
	*p*-value	0.056	0.090	0.061	0.031	0.374	<0.001	0.001
Lateral E/e′	r/rho	0.132	0.115	0.116	0.073	0.049	0.220	−0.115
	*p*-value	0.040	0.075	0.072	0.262	0.451	0.001	0.076
S′ (cm/s) *	r/rho	−0.513	−0.159	−0.480	0.092	−0.126	0.126	−0.035
	*p*-value	<0.001	0.014	<0.001	0.155	0.051	0.051	0.589
Aortic systolic diameter index *	r/rho	−0.037	−0.564	−0.044	−0.136	−0.220	0.056	0.026
	*p*-value	0.567	<0.001	0.492	0.035	0.001	0.387	0.692
Aortic diastolic diameter index *	r/rho	0.262	−0.333	0.255	−0.158	−0.161	−0.030	−0.009
	*p*-value	<0.001	<0.001	<0.001	0.014	0.012	0.644	0.892
Aortic Strain	r/rho	−0.730	−0.273	−0.697	0.092	−0.030	0.165	0.024
	*p*-value	<0.001	<0.001	<0.001	0.153	0.644	0.010	0.715
Aortic distensibility *	r/rho	−0.721	−0.274	−0.709	0.083	−0.022	0.158	0.033
	*p*-value	<0.001	<0.001	<0.001	0.197	0.733	0.014	0.608
Aortic stiffness	r/rho	0.602	0.245	0.499	−0.061	0.004	−0.101	−0.053
	*p*-value	<0.001	<0.001	<0.001	0.348	0.956	0.118	0.412

Pearson correlation analysis (correlation coefficient = r) was used for the correlation relationships between the variables marked with (*), while Spearman correlation analysis (correlation coefficient = rho) was used for the relationships between other variables.

**Table 6:** Investigation of the impact profiles of variables on disease through univariate and multivariate logistic regression (LR) analysis.

LR Analysis								
	Univariate LR						Multivariate LR	
Variables ^†^	Nagelkerke R^2^	B	*p*	OR	95% CI		Nagelkerke R^2^ = 0.30	
					Lower Limit	Upper Limit	*p*	OR
Age, years ^ɸ^	0.001	−0.008	0.728	0.992	0.945	1.040	0.036	0.933
BMI, kg/m^2 ɸ^	<0.001	0.002	0.947	1.002	0.957	1.048	0.321	0.972
Gestational week ^ɸ^	<0.001	−0.003	0.932	0.997	0.934	1.064	0.836	1.01
CAR								
<2.34 (ref)	–	–	–	–	–	–	–	–
≥2.34	0.219	2.081	<0.001	8.02	4.00	16.06	<0.001	9.17
Aortic strain ^§^							–	–
>0.338 (ref)	–	–	–	–	–	–	–	–
≤0.338	0.054	0.825	0.002	2.28	1.362	3.821	–	–
Aortic distensibility ^§^							–	–
>0.017 (ref)	–	–	–	–	–	–	–	–
≤0.017	0.026	0.585	0.03	1.79	1.059	3.047	–	–
Aortic stiffness								
<1.325 (ref)	–	–	–	–	–	–	–	–
≥1.325	0.065	0.915	<0.001	2.50	1.476	4.221	<0.001	3.72

Reference category of analysis: Control group. Abbreviations: CI = Confidence Interval, OR = Odds ratio, LR = Logistic regression, ref = Reference subcategory. † The cut-off values were obtained from ROC analysis (based on Youden’s J index). § Due to high multicollinearity, these variables were excluded from the multivariate model. ɸ While not significant in the univariate model, these variables are included in the multivariate model as potential covariates (confounders) for adjustment purposes.

## Data Availability

The datasets generated and/or analyzed during the current study are available from the corresponding author on reasonable request.

## Abbreviations

ANCOVA	Analysis of covariance
AoDD	Aortic diastolic diameter
AoSD	Aortic systolic diameter
ASE	American Society of Echocardiography
AUC	Area under the curve
BMI	Body mass index
CAR	C-reactive protein-to-albumin ratio
CBC	Complete blood count
CI	Confidence interval
CRP	C-reactive protein
DBP	Diastolic blood pressure
HALP	Hemoglobin–Albumin–Lymphocyte–Platelet score
IQR	Interquartile range
IVSd	Interventricular septum thickness
LVEDd	Left ventricular end-diastolic diameter
LVESd	Left ventricular end-systolic diameter
LVPWd	Left ventricular posterior wall thickness
MAP	Mean arterial pressure
PNI	Prognostic nutritional index
RAR	Red blood cell distribution width-to-albumin ratio
RDW	Red blood cell distribution width
ROC	Receiver operating characteristic
SBP	Systolic blood pressure
sPAP	Systolic pulmonary artery pressure
TAPSE	Tricuspid annular plane systolic excursion
TSH	Thyroid-stimulating hormone
WBC	White blood cell count
